# Diet and exercise changes following direct-to-consumer personal genomic testing

**DOI:** 10.1186/s12920-017-0258-1

**Published:** 2017-05-02

**Authors:** Daiva Elena Nielsen, Deanna Alexis Carere, Catharine Wang, J. Scott Roberts, Robert C. Green, for the PGen Study Group, Robert C. Green, Joel B. Krier, Sarah S. Kalia, Kurt D. Christensen, Daiva E. Nielsen, Lisa S. Lehmann, Peter Kraft, J. Scott Roberts, Mack T. Ruffin, Lan Q. Le, Jenny Ostergren, Wendy R. Uhlmann, Mick P. Couper, Deanna Alexis Carere, Joanna L. Mountain, Amy K. Kiefer, Glenn Braunstein, Scott D. Crawford, L. Adrienne Cupples, Clara A. Chen, Catharine Wang, Stacy W. Gray, Barbara A. Koenig, Kimberly Kaphingst, Sarah Gollust

**Affiliations:** 1 Division of Genetics, Department of Medicine, Brigham and Women’s Hospital, EC Alumnae Building, Suite 301, 41 Avenue Louis Pasteur, Boston, MA 02115 USA; 2 Harvard Medical School, EC Alumnae Building, Suite 301, 41 Avenue Louis Pasteur, Boston, MA 02115 USA; 3 Department of Pathology and Molecular Medicine, McMaster University, Hamilton, ON L8L 2X2 Canada; 4 Community Health Sciences Department, Boston University School of Public Health, Boston, MA 02118 USA; 5 Department of Health Behavior & Health Education, University of Michigan School of Public Health, Ann Arbor, MI 48109 USA; 6 Partners Personalized Medicine, EC Alumnae Building, Suite 301, 41 Avenue Louis Pasteur, Boston, MA 02115 USA; 7 Broad Institute of MIT and Harvard, Cambridge, MA 02142 USA

**Keywords:** Direct-to-consumer, Genetic testing, Health behavior, Diet, Exercise

## Abstract

**Background:**

The impacts of direct-to-consumer personal genomic testing (PGT) on health behaviors such as diet and exercise are poorly understood. Our investigation aimed to evaluate diet and exercise changes following PGT and to determine if changes were associated with genetic test results obtained from PGT.

**Methods:**

Customers of 23andMe and Pathway Genomics completed a web-based survey prior to receiving PGT results (baseline) and 6 months post-results. Fruit and vegetable intake (servings/day), and light, vigorous and strength exercise frequency (days/week) were assessed. Changes in diet and exercise were examined using paired t-tests and linear regressions. Additional analyses examined whether outcomes differed by baseline self-reported health (SRH) or content of PGT results.

**Results:**

Longitudinal data were available for 1,002 participants. Significant increases were observed for vegetable intake (mean Δ = 0.11 (95% CI = 0.05, 0.17), *p* = 0.0003) and strength exercise (Δ = 0.14 (0.03, 0.25), *p* = 0.0153). When stratified by SRH, significant increases were observed for all outcomes among lower SRH participants: fruit intake, Δ = 0.11 (0.02, 0.21), *p* = 0.0148; vegetable intake, Δ = 0.16 (0.07, 0.25), *p* = 0.0005; light exercise, Δ = 0.25 (0.03, 0.47), *p* = 0.0263; vigorous exercise, Δ = 0.23 (0.06, 0.41), *p* = 0.0097; strength exercise, Δ = 0.19 (0.01, 0.37), *p* = 0.0369. A significant change among higher SRH participants was only observed for light exercise, and in the opposite direction: Δ = -0.2468 (-0.06, -0.44), *p* = 0.0111. Genetic results were not consistently associated with any diet or exercise changes.

**Conclusions:**

The experience of PGT was associated with modest, mostly positive changes in diet and exercise. Associations were independent of genetic results from PGT.

## Background

Direct-to-consumer (DTC) personal genomic testing (PGT) services persist, despite continued concern and interventions from regulatory agencies. Most notably, the Food and Drug Administration (FDA) sent a warning letter to 23andMe, Inc. in 2013, ordering the company to cease marketing of its health-related PGT services until it received FDA approval [1]. The FDA raised concerns about the validity of the information returned to consumers and the potential for inappropriate medical actions post-PGT. However, some have argued that PGT could enhance individual health autonomy and may motivate positive lifestyle changes [2–4]. This could be particularly beneficial, as a recent analysis across four studies concluded that a healthy lifestyle reduced the relative risk of coronary artery disease by nearly 50% among those at high genetic risk [5]. In February 2015, the FDA approved the first 23andMe health report and as of October 2015 the company is returning carrier status reports (a subset of its previous offerings) to customers [6].

The first regulatory approval of DTC-PGT in the United States has reignited academic interest in the impact and utility of returning genomic information without healthcare professional involvement. One key question is whether or not PGT has the potential to motivate diet or exercise changes that could prevent lifestyle-related chronic diseases, such as heart disease and type 2 diabetes [7]. Previous studies examining this question have reported that PGT is not associated with diet or exercise changes [8, 9] or that the PGT experience generally, including the personal context in which testing is sought (but not the individual genetic risk information received), is associated with non-specific, positive health behavior changes [10]. These prior investigations have been limited by selected convenience samples [8] or the use of general survey items that did not measure specific diet or exercise variables both pre- and post-PGT [10]. In addition, not all previous work has considered baseline health status and/or disease risk perception as factors that could influence diet and exercise changes following PGT. These are important limitations because health behavior has been shown to vary by health status as well as self-perceived health [11], and it is unknown if perceptions of personal disease risk impact diet and exercise following PGT.

The Impact of Personal Genomics (PGen) Study is a longitudinal survey assessment of actual PGT customers from two companies, 23andMe, Inc. (23andMe) and Pathway Genomics Corp. (Pathway). Specific diet and exercise variables were measured both before and after PGT, and self-reported health status (SRH) and disease risk perception were also measured. Participants’ PGT results were linked to their survey responses. Here we present an analysis using PGen Study data that examines changes to self-reported fruit and vegetable intake and exercise frequency following PGT. Prior evidence suggests that DTC-PGT users are particularly motivated to undergo PGT for purposes of health improvement [10], yet commentary on DTC-PGT often assumes that its users are likely already to be in good health (i.e. the “worried well”). For this reason, we were interested not only in whether the PGT experience could motivate health behavior change, but whether its effects would differ by user health status. We hypothesized that diet and exercise would change from pre-PGT to post-PGT, and further investigated whether the changes would vary by baseline SRH, actual genetic testing results received, or disease risk perception.

## Methods

### Study design and procedures

The design, recruitment and data collection procedures in the PGen Study have been previously reported [12]. Briefly, new customers of 23andMe and Pathway were recruited online after ordering PGT between March and July 2012. Following online consent, participants were invited to three web-based surveys administered by Survey Sciences Group, LLC (now SoundRocket): at baseline (prior to receipt of results), 2 weeks, and 6 months after results were viewed. In total, 1,464 participants completed the baseline survey and were eligible for follow-up; of these, 1,046 (71.4%) and 1,042 (71.2%) submitted the 2-week and 6-month surveys, respectively. PGT results were provided by the companies and linked to individual-level survey data. The PGen Study was approved by the Partners Human Research Committee and the University of Michigan School of Public Health Institutional Review Board.

### Survey instruments

At baseline, participants reported their demographic information and SRH was assessed using a validated 5-point scale from the SF-36 Health Survey [13]. At baseline and 6 month follow-up, daily fruit and vegetable servings were assessed using a validated 2-item food frequency questionnaire [14] (responses: None, ≤1, 2, 3, 4, ≥5 servings/day) and the number of days per week of engaging in light/moderate, vigorous, and strength exercises for at least 10 min were assessed using items adapted from the validated National Health Interview Survey [15] (responses: 0–7 days/week). At 6 month follow-up, respondents were asked “Did you make any of the following health or wellness changes that were specifically motivated by your PGT results?” and they selected Yes/No for each of “Diet” and “Exercise”. Because the median time to follow-up survey initiation in the PGen Study was 6.3 months [12], most participants responded to the baseline and 6 month surveys in opposing seasons (i.e., Spring and Fall, or Summer and Winter, depending on date of PGT). While participants were not able to complete the 6 month survey early, some participants did complete and submit the 6 month survey late, and so experienced a less significant change in seasonality between surveys. To account for the fact that change in seasonality could contribute to health behavior changes, we created a variable for *significant season change* (yes/no) based on season of baseline and 6 month survey submission. Survey season was assigned according to the month of survey submission (Spring = March-May; Summer = June-August; Fall = September-November; Winter = December-February), and *significant season change* was coded as “yes” for a change to a non-consecutive season (e.g., Spring to Fall, or Summer to Winter), and as “no” for a change to a consecutive season (e.g., Spring to Summer, or Summer to Spring) or no change in season.

### Personal genomic testing results

We have previously described how genetic risk estimates were reported and how a threshold relative risk (RR) was selected for analysis in the PGen Study [16]. Briefly, 23andMe participants received a genotype-derived numeric RR estimate for each condition, compared to an individual of the same age, gender and ethnicity; and Pathway participants were assigned, based on RR, a qualitative risk category for each condition (below average risk, average risk, and three levels of above average risk). To harmonize genetic risk information across companies and analyses, a threshold RR level of 1.2 was selected to distinguish *average* or *below average* genetic risk (23andMe RR < 1.2; two lowest Pathway categories) from *above average* genetic risk (23andMe RR ≥ 1.2; three highest Pathway categories). This threshold was consistent with the reporting standards of both companies, and endorsed by PGen Study researchers as appropriate in the context of testing common, low-penetrance genetic variants.

### Genetic risk scores and risk perception

Since PGT results were linked to participants’ survey responses, we were able to examine whether specific genetic risk information was associated with changes to diet and exercise. Because diet and exercise can modify risk of cardiometabolic diseases, we first created an *additive cardiometabolic genetic risk score* based on PGT-derived risk estimates for obesity, type 2 diabetes (T2D), and coronary heart disease (CHD). This score was the sum of the number of above average PGT risk results (referred to as "elevated risk") a participant received for the three cardiometabolic conditions (i.e. either 0, 1, 2 or 3 elevated risk results). To examine the aggregate impact of all PGT-derived disease risk estimates received, we created an *additive total genetic risk score* corresponding to the number of above average (elevated) risk results received across all health conditions in a participant’s PGT report. Depending on gender, ethnicity, and DTC-PGT company, participants received risk estimates for between 25 and 29 health conditions, which included the cardiometabolic conditions mentioned above, various cancers, neurological disorders, autoimmune disorders and others (total risk score = 0-29; complete details of the conditions included in the score are reported by van der Wouden et al. [17]).

In addition to genetic risk, we examined whether perceived risk of cardiometabolic disease influenced changes to diet and exercise. At both baseline and 6 month follow-up, participants were asked to rate their perceived risk of developing obesity, T2D and CHD on a 5-point scale (*much lower than average* = 1 to *much higher than average* = 5, with *average* = 3). An additive disease risk perception score was created by summing participant responses for perceived risk of all three conditions at both baseline and follow-up. This yielded two scores that could range from 3 to 15 (one for baseline risk perception and one for follow-up risk perception). *Change in risk perception* was calculated by subtracting the follow-up score from the baseline score and thus the variable for change in risk perception could range from -12 to 12 (see Carere et al. for details of how risk perception and change were measured in the PGen Study [16]).

### Statistical analyses

Data were obtained from participants who submitted both the baseline and 6-month survey, and had available responses for the items described above. Merged data are presented for the two PGT companies because a general agreement was made between company representatives and researchers to avoid presenting company-specific data when results could be perceived as relating to market research [18]. Descriptive statistics were used to characterize baseline demographic characteristics. Two-sided paired t-tests were used to test for significant changes in each diet and exercise item in the full sample, and within subsamples stratified by SRH (poor/fair/good vs. very good/excellent). We performed linear regression with a Tukey-Kramer test for pairwise comparisons to determine if diet or exercise changes significantly differed between SRH subgroups. These models were adjusted for the specific baseline behavior (i.e. the corresponding diet or exercise variable), PGT company, age, gender, education, income, race, ethnicity (Hispanic or non-Hispanic), BMI, SRH, and season change. To assess consistency between the measured diet and exercise variables and general self-reported dietary and exercise changes, we used two-sided t-tests to compare mean changes in fruit/vegetable intake and exercise frequency across groups stratified by general self-reported changes (Yes/No diet or exercise change).

Linear regressions of change in fruit/vegetable intake and exercise frequency on the cardiometabolic genetic risk score and total genetic risk score were conducted with adjustment for the baseline behavior, PGT company, age, gender, education, income, race, ethnicity, BMI, SRH, and season change. We used similar linear regression models of diet/exercise change on change in risk perception of cardiometabolic disease, but also adjusted for baseline risk perception in addition to the covariates listed previously. Analyses were conducted using SAS software (version 9.3; SAS Institute, Cary, NC), and linear regression models were fitted using PROC GLM. Statistical significance for all analyses was set at *p* < 0.05.

## Results

From the 1042 participants who submitted both a baseline and 6-month survey, complete data required for our analyses were available from 1002 participants. Demographic characteristics of the analytic sample are reported in Table 1. Baseline diet and exercise variables did not differ between baseline responders eligible for follow-up (*n* = 1464) and the final analytic sample (*n* = 1002). Among the 1002 participants with complete data, modest, but statistically significant increases in vegetable intake and strength exercise frequency were observed (Table 2). Vegetable intake increased by an average of 0.11 servings per day and frequency of at least 10 min of strength exercise increased by an average of 0.14 days per week. When stratified by SRH, significant increases in all diet and exercise behaviors were observed among the lower SRH group, while a significant decrease in light exercise frequency was observed among the higher SRH group (average decrease in frequency of 0.25 days per week). Adjusted linear regression models with the Tukey-Kramer pairwise comparison demonstrated that only change in light exercise differed significantly between the two SRH subgroups (*p* = 0.0452). The direction of the observed change in light exercise was opposite between the two groups (i.e. increase in frequency among the lower SRH group, decrease in frequency among the higher SRH group).

**Table 1:** Baseline demographics stratified by self-reported health

Variable	Baseline responders eligible for follow-up (*n* = 1464)	Baseline responders with complete data (*n* = 1002)	Poor/Fair/Good SRH (*n* = 449)	Very Good/ Excellent SRH (n = 551)
n (%)				
Male	567 (38.7)	401 (40.0)	156 (34.7)	245 (44.5)
Race				
White	1234 (84.3)	860 (85.8)	383 (85.3)	475 (86.2)
Black	37 (2.5)	23 (2.3)	11 (2.5)	12 (2.2)
Asian	50 (3.4)	32 (3.2)	8 (1.8)	24 (4.4)
Other/Multi-Ethnic	143 (9.8)	87 (8.7)	47 (10.5)	40 (7.3)
Hispanic	81 (5.5)	50 (5.0)	25 (5.6)	25 (4.5)
Education				
Less than College	319 (21.8)	204 (20.4)	117 (26.1)	87 (15.8)
College Degree	448 (30.6)	305 (30.4)	118 (26.3)	186 (33.8)
Some Graduate Degree	513 (35.0)	359 (35.8)	162 (36.1)	196 (35.6)
Doctoral Degree	184 (12.6)	134 (13.4)	52 (11.6)	82 (14.9)
Annual Income				
< $40,000	242 (16.5)	171 (17.1)	102 (22.7)	69 (12.5)
$40,000–$69,999	272 (18.6)	183 (18.3)	84 (18.7)	98 (178)
$70,000–$99,999	288 (19.7)	205 (20.5)	96 (21.4)	108 (19.6)
$100,000–$199,999	457 (31.2)	303 (30.2)	121 (27.0)	182 (33.0)
≥ $200,000	184 (12.6)	128 (12.8)	38 (8.5)	90 (16.3)
Missing	21 (1.4)	12 (1.2)	8 (1.8)	4 (0.7)
PGT company				
23andMe	947 (64.7)	620 (61.9)	232 (51.7)	386 (70.0)
Pathway Genomics	517 (35.3)	382 (38.1)	217 (48.3)	165 (30.0)
Mean ± standard deviation (Range)				
Age	47.5 ± 15.5 (19 – 94)	46.9 ± 15.6 (19 – 94)	47.6 ± 15.1 (19 – 91)	46.2 ± 15.9 (19 – 94)
BMI	26.9 ± 6.0	26.9 ± 6.0	28.8 ± 7.1	25.3 ± 4.4
	(15.4 – 62.0)	(16.1 – 62.0)	(16.1 – 62.0)	(16.6 – 47.3)
Fruit Servings per Day (0–5+)	2.0 ± 1.1	2.0 ± 1.1	1.9 ± 1.1	2.2 ± 1.1
Vegetable Servings per Day (0–5+)	2.5 ± 1.2	2.5 ± 1.2	2.3 ± 1.1	2.7 ± 1.2
Light Exercise per Week (0–7)	3.5 ± 2.3	3.5 ± 2.3	3.3 ± 2.2	3.7 ± 2.2
Vigorous Exercise per Week (0–7)	2.3 ± 2.1	2.4 ± 2.1	1.8 ± 2.0	2.8 ± 2.1
Strength Exercise per Week (0–7)	1.4 ± 1.8	1.4 ± 1.8	1.1 ± 1.8	1.7 ± 1.8

**Table 2:** Self-reported diet and exercise changes after PGT

	Full Sample (*n* = 1,002)		Poor/Fair/Good SRH (*n* = 449)		Very Good/Excellent SRH (*n* = 551)		Tukey-Kramer adjusted *p*-value^a^
Change	%	Paired *t*-test	%	Paired *t*-test	%	Paired *t*-test	
Fruit servings per days							
Decrease	24.0	Δ = 0.06 (-0.005, 0.12) *p* = 0.07	21.2	Δ = 0.11 (0.02, 0.21) *p* = 0.0148	26.1	Δ = 0.01 (-0.07, 0.10) *p* = 0.77	0.7191
No Change	49.0		50.1		48.3		
Increase	27.0		28.7		25.6		
Vegetable servings per day							
Decrease	21.2	Δ = 0.11 (0.05, 0.17) *p* = 0.0003	19.8	Δ = 0.16 (0.07, 0.25) *p* = 0.0005	22.3	Δ = 0.06 (-0.01, 0.14) *p* = 0.11	0.7032
No Change	49.8		50.6		49.0		
Increase	29.0		29.6		28.7		
Light exercise							
Decrease	35.2	Δ = -0.02 (-0.16, 0.13) *p* = 0.80	31.2	Δ = 0.25 (0.03, 0.47) *p* = 0.0263	38.7	Δ = -0.25 (-0.06, -0.44) *p* = 0.0111	0.0452
No Change	31.0		32.3		30.0		
Increase	33.8		36.5		31.3		
Vigorous exercise							
Decrease	28.7	Δ = 0.09 (-0.02, 0.21) *p* = 0.11	24.5	Δ = 0.23 (0.06, 0.41) *p* = 0.0097	32.3	Δ = -0.02 (-0.18, 0.13) *p* = 0.76	0.1236
No Change	40.6		44.5		37.4		
Increase	30.7		31.0		30.3		
Strength exercise							
Decrease	21.6	Δ = 0.14 (0.03, 0.25) *p* = 0.0153	17.4	Δ = 0.19 (0.01, 0.37) *p* = 0.0369	25.1	Δ = 0.10 (-0.05, 0.24) *p* = 0.18	0.2690
No Change	53.2		57.2		49.7		
Increase	25.2		25.4		25.2		

^a^Adjusted for baseline behavior, company, age, gender, education, income, race, ethnicity, baseline BMI, baseline self-reported health, season change

Thirty percent of participants reported making a change to their diet that was specifically motivated by their PGT results, and 26% reported changing their exercise based on their PGT results (Table 3). The subset of respondents that reported diet changes had corresponding significant increases in intakes of fruit and vegetables (an average increase of 0.19 and 0.31 servings per day, respectively), while the subset that reported exercise changes had corresponding significant increases in the frequency of at least 10 min per day of vigorous and strength exercise (an average frequency increase of 0.58 and 0.47 days per week, respectively).

**Table 3:** Comparison of changes in diet and exercise variables to self-reported general wellness changes

Did you make any of the following health or wellness changes that were specifically motivated by your PGT results?		
Diet	Yes N = 301 (30%)	No N = 701 (70%)
Fruit Servings per Day	Δ = 0.19 (0.03, 0.31) *p* = 0.0027	Δ = 0.001 (-0.07, 0.07) *p* = 0.96
Vegetable Servings per Day	Δ = 0.31 (0.19, 0.42) *p* < 0.0001	Δ = 0.02 (-0.04, 0.09) *p* = 0.50
Exercise	Yes N = 255 (26%)	No N = 747 (74%)
Light Exercise per Week	Δ = 0.18 (-0.11, 0.47) *p* = 0.22	Δ = -0.09 (-0.26, 0.08) *p* = 0.30
Vigorous Exercise per Week	Δ = 0.58 (0.33, 0.83) *p* < 0.0001	Δ = 0.08 (-0.20, 0.05) *p* = 0.25
Strength Exercise per Week	Δ = 0.47 (0.23 0.71) *p* = 0.0002	Δ = 0.03 (-0.10, 0.15) *p* = 0.69

Summary information about the additive genetic risk score for cardiometabolic conditions and the additive genetic risk score for all health conditions is shown in Table 4. The mean number of elevated test results for the three cardiometabolic conditions was 0.48 ± 0.64 (mean ± SD). No significant associations were found between the cardiometabolic genetic risk score and change in any diet or exercise variable (Table 5). Taking all tested conditions together, the number of elevated test results ranged from 0 to 13 and the average number of elevated test results for all health conditions was 5.6 ± 2.2 (mean ± SD). The total genetic risk score was inversely associated with change in fruit servings per day (β = -0.03 (-0.06, -0.01), *p* = 0.02) (Table 5). From baseline to follow-up, the average change in overall risk perception of cardiometabolic disease increased (0.60 ± 2.2 (mean ± SD)), and this change in risk perception was inversely associated with change in vigorous exercise (β = -0.08 (-0.14, -0.02), *p* = 0.01) (Table 5). While these two effects were significant, the inclusion of genetic risk scores or perceived risk did not explain any more variance in self-reported diet and exercise changes than the covariate-only model (Table 5).

**Table 4:** Distribution of elevated risk PGT results

	Number of Elevated Results	n (%)
Cardiometabolic diseases		
	0	536 (56.9%)
	1	333 (35.3%)
	2	70 (7.4%)
	3	3 (0.3%)
All diseases (including cardiometabolic)		
	0	1 (0.1%)
	1	14 (1.5%)
	2	40 (4.3%)
	3	89 (9.5%)
	4	150 (15.9%)
	5	165 (17.5%)
	6	179 (19.0%)
	7	125 (13.3%)
	8	93 (9.9%)
	9	48 (5.1%)
	10	23 (2.4%)
	11	9 (1.0%)
	12	5 (0.5%)
	13	1 (0.1%)

**Table 5:** Impact of genetic test scores and risk perception on changes in diet and exercise

Variable	β (95% CI), *p*-value	R^2^
Cardiometabolic genetic test score		
Fruit Servings per Day	β = -0.04 (-0.14, 0.06), *p* = 0.42	0.18
Vegetable Servings per Day	β = -0.02 (-0.11, 0.08), *p* = 0.73	0.14
Light Exercise per Week	β = -0.01 (-0.22, 0.20), *p* = 0.90	0.28
Vigorous Exercise per Week	β = -0.09 (-0.26, 0.08), *p* = 0.29	0.23
Strength Exercise per Week	β = 0.02 (-0.14, 0.18), *p* = 0.82	0.23
Total genetic risk score		
Fruit Servings per Day	β = -0.03 (-0.06, -0.01), *p* = 0.02	0.18
Vegetable Servings per Day	β = -0.007 (-0.03, 0.02), *p* = 0.63	0.14
Light Exercise per Week	β = 0.01 (-0.05, 0.07), *p* = 0.73	0.28
Vigorous Exercise per Week	β = -0.03 (-0.08, 0.02), *p* = 0.28	0.23
Strength Exercise per Week	β = -0.005 (-0.05, 0.04), *p* = 0.82	0.23
Change in risk perception^a^		
Fruit Servings per Day	β = -0.004 (-0.04, 0.03), *p* = 0.81	0.20
Vegetable Servings per Day	β = 0.005 (-0.03, 0.04), *p* = 0.75	0.15
Light Exercise per Week	β = -0.04 (-0.11, 0.04), *p* = 0.31	0.28
Vigorous Exercise per Week	β = -0.08 (-0.14, -0.02), *p* = 0.01	0.26
Strength Exercise per Week	β = 0.004 (-0.06, 0.06), *p*= 0.91	0.23

Adjusted for: baseline behavior, company, season change, age, gender, education, income, race, ethnicity, baseline BMI, baseline self-reported health

β estimates are raw coefficients

^**a**^Also adjusted for baseline perceived riskNote: The presented model R^2^ values (with genetic risk score/risk perception) are all equivalent to the covariate-only R^2^ values for each fitted model

## Discussion

In a longitudinal study of actual DTC-PGT customers, we found modest, but statistically significant, increases in self-reported vegetable consumption and strength exercise frequency post-PGT. There appears to be a difference in diet and exercise changes when participants are separated by self-reported health status, as the lower SRH group also demonstrated a significant increase in fruit consumption and frequency of light and vigorous exercise, while a decrease in the frequency of light exercise was observed among the higher SRH group. Although nearly a third of participants reported making diet and exercise changes that were directly motivated by their PGT results, there was no consistent evidence that specific genetic risk information received from PGT, or post-PGT change in cardiometabolic disease risk perception, were associated with the specific diet and exercise variables that we measured. The two significant findings observed in our analyses of genetic risk information and change in risk perception deviate from an overall pattern of null results that suggest that the risk variables we evaluated were not associated with observed changes in diet and exercise following PGT.

The consistent observation of diet and exercise improvements within the lower SRH group, but not within the higher SRH group may reflect the fact that individuals with higher baseline SRH were already engaging more frequently in healthy diet and exercise behaviors before PGT; moreover, they may in fact be objectively healthier than the lower SRH group, and have a lesser (or at least perceived lesser) need to improve their diet and exercise. The apparent reduction in frequency of light exercise in this group is also of interest, for if accurate, this could represent an undesirable effect of receiving genetic risk information among individuals with high SRH. We cannot determine whether health behavior changes we observed were a consequence of the PGT experience, or if the decision to pursue PGT was part of a broader goal to improve one’s health that incorporated an intention to modify diet and exercise. Finally, although we did not find any consistent evidence that cardiometabolic or total genetic risk burden were associated with diet or exercise changes post-PGT, it is possible that some other genetic information returned by the companies (e.g., results pertaining to pharmacogenomics or non-medical physical traits) could motivate diet and exercise behavior change. In fact, it may be the case that no single genetic result is universally motivating to PGT consumers, but rather that certain individuals may be inclined to change their diet based on an elevated risk of type 2 diabetes, while others (perhaps owing to personal context or family history) may be more immediately motivated by an elevated genetic risk of breast cancer. Nevertheless, the recent report of reduced coronary events among individuals with high genetic risk who were noted to follow good lifestyle habits [5] highlights the importance of efforts to use genetic testing as a tool to motivate positive lifestyle changes.

Prior studies of the effect of genetic risk information on health behavior change have not typically reported significant post-testing changes to diet or exercise [8–10]. For example, Bloss et al. followed approximately 2000 Navigenics customers over a year and examined changes in dietary fat intake and exercise [8, 9]. In that study, no significant changes were observed at either the 3-month or 12-month follow-up; however, participants in this study were employees of a personalized medicine research institute, and had a PGT experience that was facilitated by the research team (e.g., participants could ask questions of researchers during the testing process). Kaufman et al. reported a cross-sectional post-PGT survey of 23andMe, deCODEme, and Navigenics customers, of which one third reported that they were being more careful about their diet and 14% reported they were exercising more. In addition, they found evidence that self-reported behavior change varied by self-perceived health status (e.g., the poorer self-perceived health group was more likely to report changes to supplement use) [10]. A major limitation of these findings, however, is that the data were collected at only one time point, and no specific diet or exercise behavior variables (e.g., frequency, intensity) were measured.

In addition to the observational studies described above, systematic reviews of randomized controlled trials (RCTs) and other trials have demonstrated few effects of disclosing genetic risk information on health behavior [19, 20]. (However, it is important to note that most studies examining the impact of genetic information are not comprised of PGT consumers). For example, a 2010 Cochrane review examined the effects of communicating DNA-based risk estimates on diet, physical activity and smoking cessation from 13 studies [19], and the authors concluded that the information had little or no effect on physical activity and smoking cessation, but might have a small effect on diet. A recent update to this systematic review examined 18 studies (of which 7 examined diet and 6 examined physical activity) and concluded that DNA-based risk estimates did not change any of the health behavior outcomes that were assessed [20]. However, despite this conclusion, the pooled analysis of the dietary study was borderline significant (*p* = 0.05) and the authors noted that there may be a small effect of genetic risk communication on diet. Indeed, a number of RCTs and intervention studies have demonstrated dietary changes following disclosure of genetic information [21–24]. While our investigation utilized a prospective observational design, it is worthwhile to note some consistency of results between the different research approaches. Moreover, there is likely a substantial degree of heterogeneity among both observational studies and RCTs in the specific type of genetic information that is returned, the presentation of the information, and the health-related recommendations that are given to individuals. Heterogeneity may contribute to some varied observations and effects that have been reported in the literature on this topic.

Our results support the position that DTC-PGT has the potential to motivate health behavior change in users who may benefit from diet and exercise modifications, although the small magnitude of observed diet and exercise changes (on the order, for example, of a few to a dozen additional days of exercise per year) indicates that genetic risk information – at least as provided through a commercial, DTC model – is likely limited in its power to effect change. Nonetheless, we are encouraged by the dual findings that nearly a third of participants reported making diet or exercise changes on the basis of their DTC-PGT results, and that food intake and exercise frequency measurements were consistent with reported changes, particularly among those participants who rated themselves as having lower health status. If DTC-PGT can effect behavior change, it is likely because its users are already sufficiently health-conscious, and in some cases specifically motivated to obtain testing as a means to learn about and improve their health [17, 25–28]. Therefore, rather than its consumers responding to specific genetic risk information or accompanying recommendations, it may be the case that DTC-PGT motivates behavior change via a “halo effect”: [29] participants emerge from the PGT experience (considered to begin when they first learn of commercial genetics and engage in decision-making about testing, and to continue through to their extended contemplation and sharing of results with friends, family, and health care providers) with a reminder of the importance of certain health behaviors and a motivation to play an active role in their health management. This interpretation is consistent with the fact that DTC-PGT reports contain dozens of results, contextualized within broad educational components addressing disease etiology, both genetic and non-genetic risk factors, and genetic mechanisms of disease [30]. For example, DTC-PGT reports commonly summarize the results of a prior epidemiology studies, present population disease statistics, and orient consumers to the concepts and interpretation of odds ratios and relative risk; moreover, these reports are carefully personalized to the consumer (e.g., by repeatedly using their name, referring to their gender and age, and describing their unique genetic makeup), which may make the information feel more relevant and valuable to the individual consumer. Within this enriched, educational, and highly personalized context, DTC-PGT as a health education activity may have a unique ability to impact how individuals perceive health, and how they make decisions regarding their health behaviors and medical care.

Limitations of this study include its reliance on self-reported data and observational design. However, the consistency we observed across survey items measuring similar effects (i.e. change in specific diet and exercise variables and general self-report of diet and exercise changes) is reassuring. Moreover, our study improves upon limitations of previous observational work, particularly in its measurement of pre- and post-disclosure changes to specific diet and exercise variables using validated tools, consideration of baseline health status, measurement of participants’ perceptions of their own disease risks, and our sample of customers who sought commercial PGT online [18]. While the PGen Study sample is somewhat homogeneous (e.g., largely White), there is evidence to suggest that PGen Study enrollees are broadly representative of the typical DTC-PGT consumer [12]. Our findings are not intended to be generalizable to the general U.S. population, but rather to the individuals who pursue commercial DTC-PGT. The changes we observed to diet and exercise were self-reported and just fractions of a dietary serving and exercise frequency, so the significance of these observations as they relate to health outcomes is uncertain. We also did not distinguish between variable factors such as exercise duration or type of fruit or vegetable. Moreover, it is unclear to what extent reporting may have been influenced by social desirability or persisted longer than 6 months. However, modest improvements in diet and exercise have been shown to be associated with population health [31–33]. Finally, because this was an observational study, the design does not enable us to have accounted for all factors that could have influenced our outcome variables of interest. Moreover, we cannot rule out the possibility that our findings were due to chance, particularly given the number of hypothesis tests performed. We also note that the DTC-PGT climate in the United States has changed since the PGen Study was conducted [1], and that 23andMe no longer offers consumers the disease risk estimates reported here, while Pathway Genomics has left the DTC market altogether. Thus, our findings do not accurately reflect a current product on the market, but have the advantage of capturing a consumer experience about which the FDA has requested additional research, and which may be reintroduced in the future, pending FDA approval.

## Conclusions

Our sample of PGT consumers made diet and exercise changes following PGT. These changes were independent of both cardiometabolic genetic risk and total genetic risk, and were also independent of changes in disease risk perception. As advanced genomic technologies (e.g., whole genome/exome sequencing) become more accessible to consumers, it will be important to assess whether or not these technologies have a similar impact on diet and exercise behaviors.

## Abbreviations

DTC-PGT: Direct-to-consumer personal genomic testing
FDA: United States Food and Drug Administration
PGT: Personal genomic testing
SRH: Self-reported health
